# Soluble Aβ42 Acts as Allosteric Activator of the Core Cholinergic Enzyme Choline Acetyltransferase

**DOI:** 10.3389/fnmol.2018.00327

**Published:** 2018-09-13

**Authors:** Amit Kumar, Erica Lana, Rajnish Kumar, Christina Unger Lithner, Taher Darreh-Shori

**Affiliations:** Division of Clinical Geriatrics, Center for Alzheimer Research, Department of Neurobiology, Care Sciences and Society, Karolinska Institutet, Stockholm, Sweden

**Keywords:** β-amyloid, choline acetyltransferase, cholinergic system, Alzheimer’s disease, enzyme kinetics

## Abstract

Two major questions in the field of Alzheimer-type dementia remain elusive. One is the native function of amyloid-β (Aβ) peptides and the other is an early deficit in the central cholinergic network. Nevertheless, recent evidence suggests that Aβ peptides are involved in the regulation of acetylcholine (ACh) homeostasis either by allosteric activation of ACh-degrading cholinesterases or by inhibiting the high-affinity choline uptake transporter. In the current study, we report that Aβ peptides, in particular Aβ42, allosterically enhances the catalytic rate of the core-cholinergic enzyme choline acetyltransferase (ChAT), responsible for biosynthesis of ACh. Detailed *in vitro* enzyme kinetic analysis indicated that both soluble Aβ40 and Aβ42 enhanced the catalytic efficiency of ChAT by ∼21% and 26% at physiological concentration ranges found in human cerebrospinal fluid (CSF). Further analyses indicated that activation of ChAT by Aβ was highly specific. Intriguingly, Aβ42 exhibited an EC_50_ of activation potency at 10-fold lower concentrations compared to Aβ40. The activation was persistent even in the presence of a physiological Aβ 40/42 mixture ratio, expected in human CSF. In conclusion, we report for the first time that Aβ42 peptide acts as allosteric enhancers of ACh-biosynthesizing enzyme ChAT. Together with two previous observations, this points to a complex molecular cross-talk between Aβ and the enzymatic machinery involved in maintaining cellular, synaptic and extra-synaptic ACh homeostasis, warranting further investigation.

## Introduction

Alzheimer’s disease (AD) is the most dominant form of dementia, affecting more than 47 million people worldwide, and this number is projected to increase fourfold by 2050 (World Health Organization [WHO], 2015). Despite decades of research, the exact cause of AD is still unknown. However, research findings during the last decades have put amyloid-β (Aβ) peptide, generated by proteolytic processing of amyloid-precursor protein (APP), in the center of AD etiology (Selkoe, 2000, 2001). Aβ peptides can undergo self-association to form multimeric proteinaceous aggregates ranging from small soluble oligomers (∼2–50mers) to large insoluble fibrils, which can deposit in the AD brain as senile plaques (Selkoe, 2001). Moreover, recent studies have suggested Aβ oligomers as the primary species instigating the symptoms of AD way before the formation of senile plaques (Hartley et al., 1999; McLean et al., 1999; Mucke et al., 2000; Lesne et al., 2006; Shankar et al., 2008; Tomiyama et al., 2010). Besides Aβ deposition, other pathological changes in AD include dysfunction of cholinergic neurons and aggregation and deposition of hyperphosphorylated tau protein as neurofibrillary tangles (Brion, 1998). However, the exact knowledge of the underlying mechanism connecting all these pathological changes in AD is still lacking.

Cholinergic neurons are defined by the expression of the enzyme choline acetyltransferase (ChAT; EC 2.3.1.6). ChAT is the main enzyme involved in the biosynthesis of the key neurotransmitter acetylcholine (ACh) from choline and acetyl-CoA (ACoA). On the contrary, the enzymes acetylcholinesterase (AChE) and butyrylcholinesterase (BuChE) break down ACh and are expressed by the cholinoceptive cells/neurons. The central cholinergic neuronal network operates in close association with different cognitive domains of the brain that are involved in memory, learning, and attention and is highly vulnerable to the effects of Aβ aggregates (Selkoe, 2000). Moreover, in the AD brain, the dysfunction and loss of cholinergic neurons of the basal forebrain regions and their projections to hippocampus and neocortex regions correlate well with cognitive decline (Price, 1986; DeKosky and Scheff, 1990). The cholinergic neuronal loss is further accompanied by reduced high-affinity choline uptake (HACU) and ChAT activity (Rylett et al., 1983; Price, 1986; Wurtman, 1992; Quirion, 1993).

The native function of the Aβ peptides, apart from their main involvement in AD pathology, is still unresolved. However, emerging reports indicate that they might be involved in the modulation of a diverse range of signaling pathways and protein kinases (Combs et al., 1999; Dineley et al., 2001; Wyss-Coray et al., 2001).

The functional relationship between Aβ and the cholinergic system, especially ChAT, is quite complex and still need further exploration to gain a better understanding of Aβ native function and role in AD pathology. Some reports suggest that soluble/oligomeric Aβ (in picomolar to nanomolar concentrations) upon short-term exposure can affect both ACh synthesis and K^+^-mediated ACh release from rat brain hippocampal slices or neuronal cell cultures, leading to presynaptic cholinergic dysfunction (Kar et al., 1996, 1998; Pedersen et al., 1996; Hoshi et al., 1997; Pedersen and Blusztajn, 1997; Satoh et al., 2001; Nunes-Tavares et al., 2012). Several independent studies reported about 40–50% reduction in ACh synthesis in cultured cholinergic neurons upon exposure to a high nanomolar concentration of Aβ42 (Pedersen et al., 1996; Hoshi et al., 1997; Kar et al., 1998; Nunes-Tavares et al., 2012). Animal studies also suggest that prolonged exposure to Aβ peptides results in neuronal degeneration/cognitive decline in both rat and mice models (Nitta et al., 1994; Giovannelli et al., 1995; Harkany et al., 1995; Itoh et al., 1996; Maurice et al., 1996).

However, in none of these studies the inhibitory effect of Aβ peptides on ACh synthesis can be linked to a direct inhibition of ChAT activity. Interestingly, some more recent studies have indicated that Aβ peptides act directly as allosteric modulators of cholinergic signaling by forming highly stable and soluble complexes with apolipoprotein-E and cholinesterases, which are termed BAβACs (Darreh-Shori et al., 2011a,b; Vijayaraghavan et al., 2013; Kumar et al., 2016b). In BAβACs, Aβ promotes hyper-activation of cholinesterases, and thereby causes an increased ACh degradation.

Thus, a direct mechanistic study on whether Aβ peptides also directly interact with ChAT and regulate its activity is still lacking. In this study, we report for the first time that Aβ peptides interact directly with human ChAT protein *in vitro*. We show that this interaction occurs at physiologically relevant concentrations of Aβ40, Aβ42, and of a mixture of both peptides. By using a newly designed high-throughput fluorometric assay, which allows direct kinetic measurement of ChAT activity, we show that Aβ peptides, in particular Aβ42, increase the activity of ChAT. Additionally, we determined the half-maximal response (EC_50_) values to elucidate the effect of Aβ peptides on catalytic efficiency of human recombinant ChAT (rChAT) protein, by performing detailed *in vitro* enzyme kinetic studies.

## Materials and Methods

### Purification of Recombinant ChAT

Recombinant ChAT was purified as described previously (Kumar et al., 2016a, 2017). Briefly, DYT media was inoculated with an O/N culture of *E. coli* BL21 Rosetta2 transformed with pProExHTa-ChAT (a gift from Dr. Brian Shilton, Department of Biochemistry, University of Western Ontario, London, ON, Canada). The bacteria were grown in shaking incubator at 37°C with 200 rpm until the optical density at 600 nm reached 0.5. After which, 0.5 mM IPTG was added and His_6_-ChAT was expressed for approximately 16 h at 18°C. Next, the cells were harvested and His_6_-ChAT was purified with “Ni-NTA fast start Kit” (Qiagen) following the manufacturer’s instructions. The elution buffer was exchanged to storage buffer [10 mM Tris pH 7.4, 500 mM NaCl, 10% (v/v) glycerol] using 30 kDa molecular weight cutoff Amicon Ultra concentrators (Merck Millipore). The purity and molecular weight of the protein were determined using SDS-PAGE electrophoresis. The total protein concentration was determined using DC Protein Assay (Bio-Rad). The purified protein was aliquoted and stored at −80°C.

### Novel Fluorometric *in vitro* ChAT Activity Measurement Assay

ChAT activity was measured with our newly developed robust in-house fluorometric assay, using human rChAT protein. Aβ peptides were purchased from rPeptide (Aβ40 Cat no. A-1153-1 and Aβ42 Cat no. A-1166-1) and 100 μM stocks were prepared in DMSO, as described before (Kumar et al., 2016b) and stored in small aliquots at −80°C. The reagents, choline chloride (C7017), acetyl coenzyme-A (ACoA, A2181), 7-diethylamino-3-(4-maleimidophenyl)-4-methylcoumarin (CPM) (C1484), thioflavin-T (ThT; T3516), and gelatin (G7041) were purchased from Sigma-Aldrich (St. Louis, MO, United States).

The ChAT assay can be run in either 96-well (as done in the current work) or 384-well plates with a CV of less than 5%. In order to test the assay robustness and suitability for performing measurement of ChAT activity in presence of soluble Aβ peptides, 50 μL/well of the rChAT (0.146 μg/mL final concentration) was incubated in 96-well plates (Nunc^TM^) with 50 μL/well of recombinant Aβ (6.4 ng/mL Aβ40, or 0.16 ng/mL Aβ42, or alternatively a mixture Aβ40:Aβ42 at ratio 10:1 consisting of 6.4 ng/mL and 0.64 ng/mL final concentrations, respectively), in presence of 150 μM choline chloride (50 μL/well) for 30 min at room temperature with orbital shaking, in dilution buffer [10 mM Tris base, 150 mM NaCl, 1.0 mM EDTA, 0.05% (w/v) Triton X-100; pH 7.4] with or without 1 mg/mL gelatin. Then, 50 μL of a cocktail-A [dilution buffer containing 10 μM ACoA (final concentration) and 15 μM CPM (final concentration)] was added to each well. Immediately after adding the cocktail-A, the changes in fluorescence were monitored kinetically at 479 nm emission wavelength after exciting at 390 nm wavelength, at 1- to 2-min intervals for 15–20 min using a microplate spectrophotometer reader (Infinite M1000, Tecan).

Each Aβ peptide dilution was run in triplicates. On each 96-wells plate, several enzyme wells without Aβ were also included as positive controls and for estimating the activation level by Aβ peptides. Negative controls were wells without enzyme, but with all the other above-mentioned reagents. The kinetic rate of enzyme activity (as ΔRFU/min slope data) was calculated and processed using the GraphPad Prism 7 analysis software. The actual rate values as well as statistical analysis values (*P*-values) of ChAT activity at different Aβ concentrations or alone were calculated after fitting the curve with the linear regression function in the GraphPad Prism 7.

Additional assay control tests were performed for checking for any Aβ interference on the assay detection system by incubating increasing concentrations of Aβ40 and Aβ42 (1:5 dilutions; 0–800 ng/mL for Aβ40; 0–100 ng/mL for Aβ42) with different combinations of the assay reagents (Aβ+choline+CPM+AcCoA; Aβ+choline+CPM; Aβ+choline+AcCoA), in absence of ChAT, and checking for any change in the fluorometric measurements.

Assay tests of ChAT activity, without Aβ, in presence of different DMSO concentrations (0.167% and 1.67%) were also performed, to confirm that the low DMSO content present in the experiment wells, due to carryover from Aβ stock solutions, was in an optimal range that did not affect ChAT activity measurements. Moreover, to avoid any bias, for all the experiments of this work, the DMSO content in the wells of the same assay plate was kept uniform by addition of DMSO in the buffer for those wells that did not contain Aβ.

Last, an assay control test with increasing Aβ concentrations (ranging from 0 to 160 ng/mL) in presence of ChAT but in absence of the enzyme substrate choline was also performed, to check whether ChAT would be able to utilize Aβ as substrate, and discard the possibility that any measured fluctuation in ChAT activity could be due to this occurrence.

### Measurement of EC_50_ Values of the Aβ Peptides for ChAT Activation

To study the effect of different physiological concentrations of Aβ40 and Aβ42 on ChAT activity, as well as to calculate the EC_50_ values for the peptides, the above-described *in vitro* fluorometric ChAT activity assay protocol was followed. To encompass the physiological concentration range of Aβ peptides, dilution series consisting of eight different Aβ40 and Aβ42 concentrations were prepared, ranging from 0 to 800 ng/mL for Aβ40 and from 0 to 100 ng/mL for Aβ42 (1:5 dilution steps).

The final DMSO concentration in the wells was kept below 2%, as we have shown previously that concentrations ≤5%, do not affect the ChAT assay (Kumar and Darreh-Shori, 2017; Kumar et al., 2017).

In addition, to study the effect of very high concentrations of Aβ40 and Aβ42 on ChAT activity, experiments with a different dilution series were performed, using eight concentrations ranging from 0 to 3,800 ng/mL (1:2 dilutions) for both peptides.

Each Aβ peptide dilution was run at least in triplicate-wells. Two or more independent replicates of the experiments were performed.

The kinetic rate of enzyme activity (as ΔRFU/min slope data) was calculated and the % rate at each specified concentration of Aβ peptides was further calculated, using the activity of the positive control wells (with no Aβ) as the baseline reference. The ChAT activity in the control wells was considered as 100% during analyses. The EC_50_ values were calculated after fitting the curves with non-linear regression log concentration of agonist vs. response functions of GraphPad Prism 7.

### Measurement of the Combined Effect of Aβ Peptides (Aβ40:Aβ42 Mixture), Within the Physiological Concentration Range, on ChAT Activity

*In vivo* both Aβ40 and Aβ42 exist together. In cerebrospinal fluid (CSF) of AD patients, for instance, the concentration of these peptides is about 4,000 pg/mL and 400 pg/mL, respectively. The concentration of these peptides is expected about twice as much in CSF of control subjects. Thus, the ratio of Aβ40:Aβ42 in human CSF is about 10:1 (Kumar et al., 2016b). For these studies, we hence prepared dilution series consisting of eight different mixture concentrations of Aβ40:Aβ42, encompassing a range from 0 to 800 ng/mL of Aβ40 and 0 to 80 ng/mL Aβ42 to mimic the *in vivo* physiological concentration range of Aβ peptides. The final DMSO concentration in the wells was kept below 2%. Each Aβ mixture dilution was run at least in triplicates. Three independent replicates of the experiment were performed. As before, on each 96-wells plate, several enzyme wells without Aβ were also included as positive controls and reference values. Negative controls were wells without the enzyme, but with the other additives.

The kinetic rate of ChAT activity (as ΔRFU/min slope data) was calculated and processed using GraphPad Prism 7. The % rate of ChAT activity at each specified Aβ40:Aβ42 concentration was also calculated, using the enzyme activity in the control wells (with no Aβ) as the 100% reference value. The EC_50_ values were calculated after fitting the curves with non-linear regression log of (agonist) concentrations vs. response function in GraphPad Prism 7.

### Thioflavin-T (THT) Fluorescence Aβ Fibrillization Assay

The ThT fluorescence assay was used to ensure that the Aβ peptides remained soluble during the aforementioned experimental setups. The ThT fluorescence assay was performed in 96-well black plates with a transparent bottom, as described before (Kumar et al., 2016b). Briefly, 100 μL/well of 800 ng/mL Aβ40 and 100 ng/mL Aβ42 (final concentrations) in dilution buffer [10 mM Tris base, 150 mM NaCl, 1.0 mM EDTA, 0.05% (w/v) Triton X-100; pH 7.4] were used. The concentration of ChAT, choline, and ACoA were as used in the aforementioned experiments. To each well, 100 μL/well of a 1 μM ThT solution was added, the plate was then carefully sealed and the changes in fluorescence were monitored from the bottom of the wells, at an emission and excitation wavelengths of 485 nm and 450 nm, respectively, at 15-min intervals at 37°C for an overnight duration, using a microplate spectrophotometer reader (Infinite M1000, Tecan). Each Aβ peptide concentration was run in quadruplicate. Several wells with Aβ alone were also included as control, and wells without ThT were used as negative controls.

### Statistical Analysis

The differences in the ChAT catalytic rate were statistically examined by comparisons of the slopes of the regressions lines for each condition, using the ANOVA function in GraphPad Prism 7. One-way ANOVA analysis was performed to compare changes caused by different concentrations of Aβ40 or Aβ42 in comparison with enzyme activity in the absence of Aβ peptides. An ANOVA *p*-value of less than 0.05 was considered significant, followed by Fisher’s least significant difference (LSD) *post hoc* test to compare each Aβ concentration with no Aβ condition.

## Results

### Real-Time Enzyme Kinetic Assessment Reveals Robust *in vitro* Aβ Induced Increase in ChAT Activity

To unveil the physiological and pathological influence of Aβ on the cholinergic system, we investigated whether and to which extent the activity of the core-cholinergic enzyme ChAT is modulated by direct interaction with Aβ peptides (Aβ40 and Aβ42 species, at different concentrations) by using a newly designed high-throughput real-time fluorometric assay of ChAT activity.

In order to test the robustness and suitability of the assay for measuring ChAT activity in presence of soluble Aβ, we performed pilot experiments by incubating ChAT with selected physiological concentrations of Aβ peptides and monitored the real-time changes in the enzymatic activity.

**FIGURE 1:**
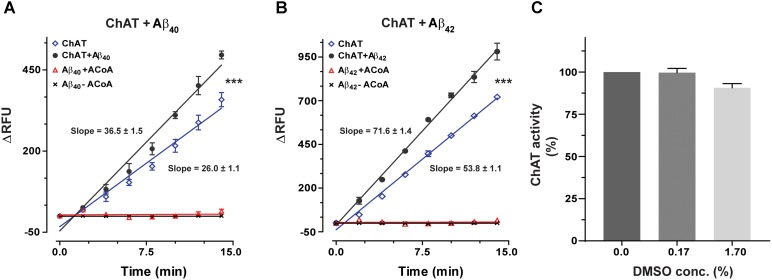
Novel real-time kinetic ChAT assay for direct monitoring of the enzyme catalytic activity. ChAT was incubated with physiological concentrations of Aβ peptides in 96-well plates and real-time changes in ChAT activity were monitored using our newly designed fluorometric assay, as described in the “Materials and Methods” section. The graphs in **(A,B)** show the measured rate of ChAT (ΔRFU/min) in the wells in the presence and absence of 6.4 ng/mL Aβ40 and 0.16 ng/mL Aβ42, respectively. ChAT activity showed a rate of 36.5 ± 1.48 ΔRFU/min and 71.6 ± 1.41 ΔRFU/min in the presence of Aβ40 and Aβ42, respectively. These slopes were statistically significant compared to slopes of ChAT activity for control, i.e., in the absence of Aβ40 (26.1 ± 1.1 ΔRFU/min) and Aβ42 (53.8 ± 1.1 ΔRFU/min). The values are shown as mean ± SD of ≥2 readings. ^∗∗∗^*p*-value < 0.0001 compared to control (no Aβ). **(C)** The effect of different concentrations (0.167% and 1.67%) of DMSO on ChAT activity was also monitored to ensure that the low DMSO content in experimental wells, resulting from diluting Aβ stocks, does not interfere with the ChAT assay. The analysis indicated that DMSO had no significant effect on ChAT activity. The values are shown as mean ± SEM of three readings.

ChAT activity showed a rate (ΔRFU/min) of 36.5 ± 1.48 and 71.6 ± 1.41 in the presence of Aβ40 (6.4 ng/mL) and Aβ42 (0.16 ng/mL), respectively (**Figures 1A,B**). These slopes were statistically significant as compared to the respective rates of 26.06 ± 1.07 (*F* = 33.21, *p* < 0.0001, **Figure 1A**) and 53.8 ± 1.11 (*F* = 98.5, *p* < 0.0001, **Figure 1B**) for the control conditions, i.e., ChAT alone.

Additional control tests of the assay were also performed, by incubating increasing concentrations of Aβ with different combinations of the other reagents of the assay or in the absence of ChAT (**Figures 1A,B**). These tests did not reveal any changes in fluorometric measurements at different Aβ concentrations, confirming that the use of Aβ peptides *per se* did not interfere with the assay detection system itself nor had they any activity by themselves (**Figure 1**). Assay tests of ChAT activity in presence of increasing DMSO concentrations were also performed to control for possible carryover of DMSO from Aβ stock solution (**Figure 1C**). DMSO had no significant effect on the ChAT activity at the concentrations used in the assays.

### Physiological Concentrations of Aβ Peptides, in Particular, Aβ42, Increase ChAT Activity *in vitro*

**FIGURE 2:**
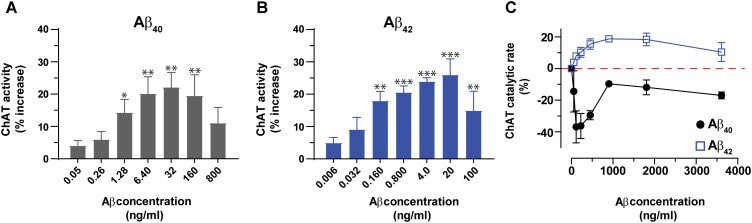
Aβ peptides directly alter the activity of ChAT at both physiological and non-physiological concentrations. ChAT was incubated with Aβ peptides at physiological concentration ranges and changes in its activity were monitored using the kinetic ChAT fluorometric assay. **(A)** The percent changes in the rate of ChAT (ΔRFU/h) by Aβ40 at the specified concentration range of 0–800 ng/mL. **(B)** The corresponding percent of changes in ChAT activity by Aβ42, at the specified concentration range of 0–100 ng/mL. The activity of control sample (ChAT with no Aβ) was considered as 100% to calculate the % increase in ChAT activity in the presence of different Aβ concentrations. The values are shown as mean ± SEM of ≥3 independent experiments. **(C)** The effect of high concentrations (non-physiological range) of Aβ peptides on ChAT activity was also monitored. ChAT was incubated with different concentrations of Aβ40 and Aβ42 peptides, ranging from 0 to 3,800 ng/mL in 96-well plates. The values are shown as mean ± SEM of two independent experiments. For Aβ40 **(A)**, the symbols ^∗^ and ^∗∗^ represents *p*-values <0.03 and <0.005 relative to the control (no Aβ40), respectively. For Aβ42 **(B)**, the symbols ^∗∗^ and ^∗∗∗^ represents *p*-values <0.005 and <0.0003, respectively, relative to the control (no Aβ42). The comparisons are based on one-way ANOVA analysis and Fisher’s LSD *post hoc* test.

Next, we expanded the pilot experiment and investigated in more detail the modulation of ChAT activity by Aβ peptides at a broad concentration range of Aβ40 and Aβ42, encompassing the possible physiological concentrations representative of CSF Aβ concentrations of patients with AD (notably ∼4 and 0.4 ng/mL for Aβ40 and Aβ42, respectively) and of healthy controls (∼6.4 and 0.8 ng/mL for Aβ40 and Aβ42, respectively). In addition, we used much higher concentration of Aβ40 (32–800 ng/mL) and Aβ42 (4–100 ng/mL) to also cover the concentration ranges used by others (Giovannelli et al., 1995; Itoh et al., 1996; Satoh et al., 2001; Nunes-Tavares et al., 2012). These analyses indicated that both Aβ40 and Aβ42 were able to increase the activity of ChAT at physiological concentrations (**Figures 2A,B**). The results show that the increase in ChAT activity reaches a plateau around 20 ng/mL for Aβ42 and 32 ng/mL for Aβ40.

However, at the higher Aβ concentration points (160 and 800 ng/mL for Aβ40; 100 ng/mL for Aβ42), the increases in ChAT activity started to decline, though the activity still remained higher than baseline values (**Figures 2A,B**).

To address this effect we repeated the analysis at a different and broader window of Aβ concentrations, namely using eight concentrations ranging from 0 to 3,800 ng/mL (equivalent to 0–833.3 nM) for both peptides (**Figure 2C**).

Interestingly, within this high concentration ranges, we observed distinct concentration-dependent effect for Aβ42 as compared to Aβ40 on ChAT activity. The results indicated that for Aβ42 peptides the effect was an activation of ChAT, although the percentage of activity increase was different from that observed at lower Aβ42 concentrations. The maximum increase of ChAT activity in this experiment was around 20%. In contrast, Aβ40 peptides induced inhibition of ChAT rather than activation, through the whole range of tested Aβ concentrations. This was unexpected considering that this effect persisted even at lower concentrations, where we found that Aβ40 activated ChAT (compare **Figures 2A,C**). Although the reason for this inconsistent behavior of Aβ40 is not clear, it could reflect an issue concerning the preparation of the dilution series of the Aβ40 solution, starting from a concentration of ∼3,800 ng/mL.

Overall, we could conclude that Aβ42 produced the most consistent effect on ChAT activity.

### Aβ42 Exhibits 10-Fold Less EC_50_ for ChAT Than Aβ40 Peptide

**FIGURE 3:**
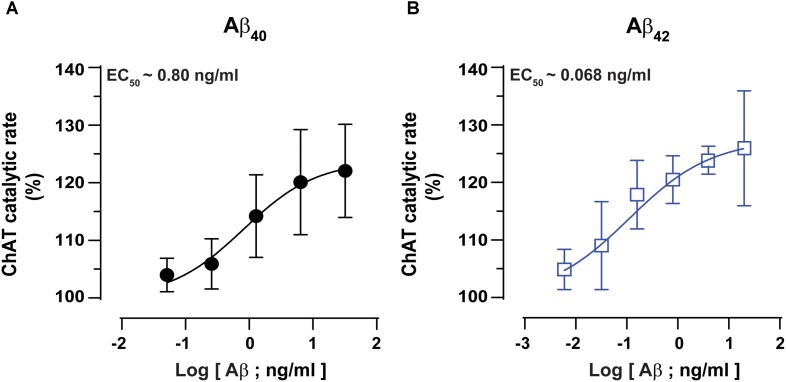
ChAT enzyme kinetic analyses for the estimation of half-maximal effective (EC_50_) concentration of Aβ40 and Aβ42. The curves in **(A,B)** represent the percent changes in the rate of ChAT (ΔRFU/h) at different Aβ40 and Aβ42 concentrations, ranging from 0 to 32 ng/mL and 0 to 20 ng/mL, respectively. The activity of control sample (ChAT with no Aβ) was considered as 100%. The EC_50_ values were calculated after fitting the curves using non-linear regression analysis in GraphPad Prism 7. The values are shown as mean ± SD of ≥3 independent experiments.

Next, we performed detailed kinetic analysis of the data shown in **Figures 2A,B** and calculated EC_50_ values for both Aβ40 and Aβ42 by fitting the ChAT activity-Aβ concentration curve (in the Aβ42: 0–20 ng/mL and Aβ40: 0–32 ng/mL ranges), using the non-linear regression analyses in GraphPad Prism 7 (**Figures 3A,B**).

These analyses indicated that Aβ42 had an EC_50_ value of ∼0.07 ng/mL, which is 10-fold less as compared to the corresponding EC_50_ of ∼0.8 ng/mL for Aβ40. Thus, we may conclude that Aβ42 peptide is a much more efficient activator of ChAT catalytic efficiency as compared to Aβ40 (**Figure 3**). The results further confirm the observation that indeed there is a fundamental difference in effects produced by Aβ40 and Aβ42 on ChAT activity, as noted in **Figure 2C**.

### The Effect of Aβ Peptides on the Catalytic Efficiency of ChAT Is Highly Specific

**FIGURE 4:**
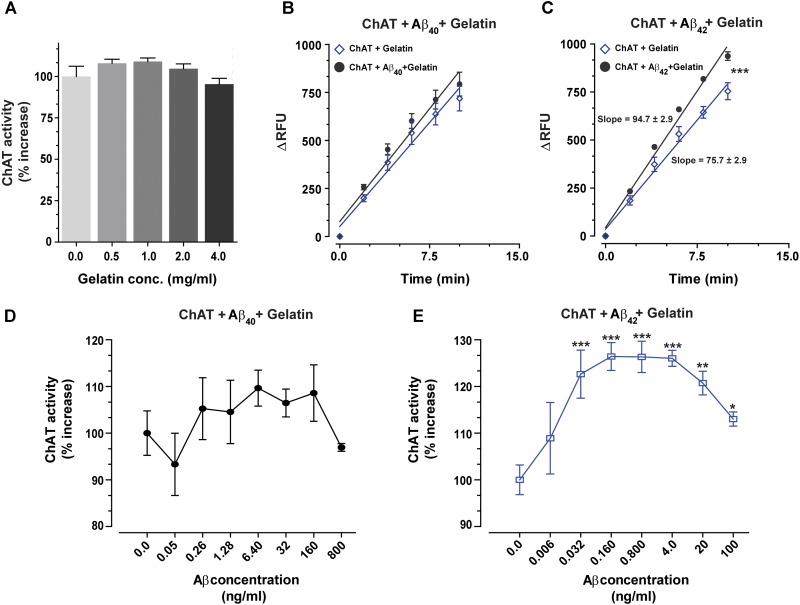
The enhanced catalytic rate of ChAT by Aβ42 is specific. **(A)** To demonstrate that Aβ-enhanced ChAT activity is a specific phenomenon, ChAT activity was first monitored at various concentrations of gelatin, ranging from 0 to 4 mg/mL. The result indicated that gelatin had no significant effect on ChAT activity. The values are shown as mean ± SD of four readings. **(B)** The kinetic rate of ChAT activity (ΔRFU/min) was measured in the presence of gelatin alone (1 mg/mL) as a control and gelatin+Aβ40 (6.4 ng/mL). Although analyses of the slopes were not statistically significant, the comparison of the elevations or intercepts was significant (*F* = 9.2, *p* < 0.004). **(C)** Then the kinetic rate of ChAT activity (ΔRFU/min) is shown in the presence of gelatin alone (control) and gelatin+Aβ42 (0.16 ng/mL). In this case, the differences in the slopes were statistically highly significant (*F* = 21, ^∗∗∗^*p* < 0.0001). The analyses of the slopes were done by using linear regression function in GraphPad Prism 7. The values are shown as mean ± SD of ≥2 readings. **(D)** Illustrates the percent changes in the ChAT activity in presence of gelatin (1 mg/mL) plus increasing concentrations of Aβ40. **(E)** Shows the corresponding percent changes in the ChAT activity in presence of gelatin (1 mg/mL) plus increasing concentrations of Aβ42. The symbols ^∗^, ^∗∗^, and ^∗∗∗^ represents *p*-values <0.03, <0.001, and <0.0005, respectively, relative to the control (no Aβ). The comparisons are based on one-way ANOVA analysis and Fisher’s LSD *post hoc* test.

The biological matrices present *in vivo*, such as in cytoplasm, ISF, CSF or plasma consist of a mixture of thousands of different proteins. To somewhat mimic these conditions and to ensure that the interactions between Aβ peptides and ChAT are specific, we repeated the analyses in the presence of gelatin concentrations ranging from 0.5 to 4 mg/mL. The changes in ChAT activity ranged from −5% to +9% (**Figure 4A**), and there was a slight increase in the rate of ChAT activity within the 0.5–2 mg/mL gelatin concentration range, which most likely reflects a stabilization of the tertiary structure of the recombinant protein. For further analysis of the effect of Aβ on ChAT activity, we hence used the concentration of 1.0 mg/mL of gelatin since it apparently had the highest stabilization effect on the activity of the rChAT protein (as appreciable from **Figure 4A**).

The result indicated that ChAT activity (ΔRFU/min) was increased by Aβ peptides even in the presence of 1.0 mg/mL gelatin. A comparison of ChAT catalytic rate at selected physiological concentrations of Aβ peptides, i.e., 6.4 ng/mL Aβ40 and 0.16 ng/mL Aβ42 are shown in **Figures 4B,C**, respectively. The overall analyses of the effect of various Aβ concentrations on ChAT activity are shown in the **Figures 4D,E**. These analyses further reinforced the observation that Aβ42 is a most efficient activator of ChAT (compare **Figure 4D** with **Figure 4E**).

### Fixed-Ratio Mixture (10:1) of Aβ40:Aβ42, Within the Physiological Concentration Range, Increases the Catalytic Efficiency of ChAT

**FIGURE 5:**
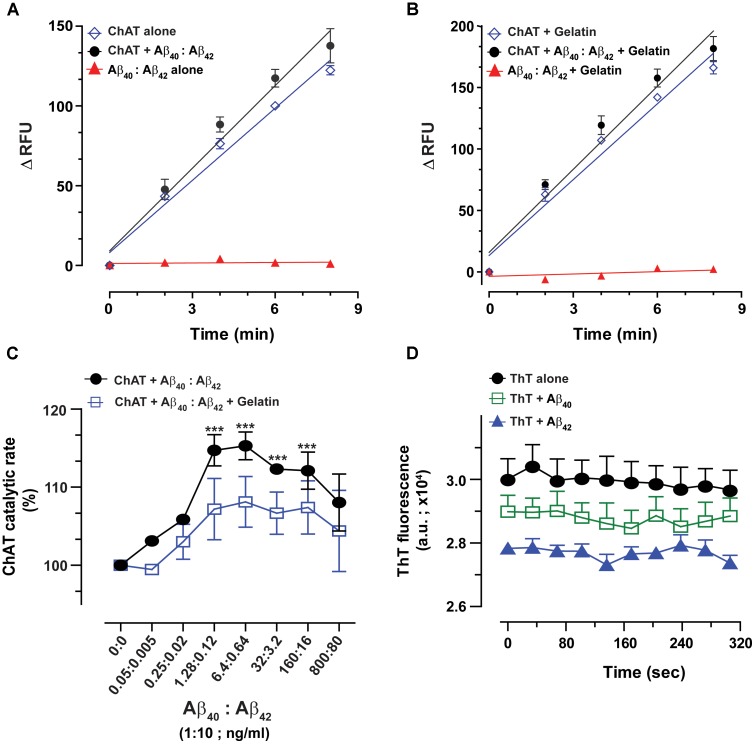
The enhanced catalytic efficiency of ChAT is persistent at a physiological ratio of Aβ40:Aβ42 mixture. Both Aβ40 and Aβ42 are present simultaneously in biological fluids at a 10:1 ratio. Therefore, we measured the ChAT activity in the presence of various concentrations of mixtures of Aβ peptides to somewhat mimic the *in vivo* conditions. The graphs **(A,B)** illustrate the kinetic rate of ChAT activity (ΔRFU/min) alone or in the presence of a mixture of Aβ40/42 (6.4/0.64 ng/mL) with and without 1 mg/mL gelatin, respectively. The data is fitted using linear regression function of the GraphPad Prism 7. The values are shown as mean ± SD of ≥2 readings. Graph **(C)** shows the percent changes in ChAT activity at the specified Aβ mixture concentrations with or without gelatin. The activity of control sample (ChAT with no Aβ) was considered as 100%. The values are shown as mean ± SEM of ≥2 independent experiments. ^∗∗∗^*p* < 0.0001, relative to the control (no Aβ), based on one-way ANOVA analysis and Fisher’s LSD *post hoc* test. **(D)** To ensure that the soluble Aβ peptides were responsible for ChAT activation, we monitored the degree of Aβ fibrillization at highest experimental concentrations (800 ng/mL Aβ40 and 100 ng/mL Aβ42) using Thioflavin-T (ThT) assay. No significant difference in the ThT fluorescence intensity was observed between Aβ containing wells and control wells (no Aβ). The values are shown as mean ± SEM of three readings.

Further, to mimic the *in vivo* conditions, where both Aβ40 and Aβ42 are present together, we repeated the analyses using a mixture of Aβ40 and Aβ42 peptides (10:1 ratio; 6.4:0.64 ng/mL). This ratio and concentration ranges were chosen to cover the relative levels of these peptides that are expected in the CSF of healthy individuals and/or AD patients. Representative curves of ChAT kinetic rate in presence of the Aβ40/42 mixture, in both presence and absence of gelatin, are shown in **Figures 5A,B**. As before, we found a significant increase in the catalytic rate of ChAT in the presence of Aβ peptides, compared to the control wells, and this held true both in presence and absence of 1 mg/mL gelatin (**Figures 5A,B**, respectively). Once again, these analyses indicated that Aβ alone or together with gelatin produced no discernable changes in the fluorescence intensity (**Figures 5A,B**).

The overall ChAT activity versus Aβ mixture concentration curve is shown in **Figure 5C**. Intriguingly, we found that the highest level of ChAT activation was achieved in presence of Aβ physiological concentration ranges (1.3–6.4 ng/mL and 0.12–0.64 ng/mL for Aβ40 and Aβ42, respectively).

Nonetheless, in the presence of gelatin, the activation level of ChAT was generally lower than the levels observed in the absence of gelatin. This could be explained by a possible fluorescence quenching effect that may occur in the assay wells due to high protein content in the assay. Despite this possible quenching effect, the overall ChAT activation pattern was essentially identical in the presence and absence of gelatin (**Figure 5C**). This provides additional assurance for the specificity of ChAT activation effect by Aβ peptides.

We further calculated the EC_50_ values by using non-linear regression analyses. Based on these analyses, Aβ40 exhibited an EC_50_ of 0.11 ng/mL in the presence of gelatin and an EC50 of 0.075 ng/mL in the absence of gelatin. The corresponding EC_50_ values for Aβ42 were 0.011 ng/mL and 0.0076 ng/mL in the presence and absence of gelatin, respectively. More importantly, these analyses indicated that the EC_50_ values for Aβ40 and Aβ42 in the mixture were ∼10-fold smaller than the EC_50_ values calculated for each peptide separately (compare to **Figure 3**).

### Soluble Aβ Peptides Are Responsible for the Activation of ChAT

To ensure that the ChAT activation effect we are observing in our study is due to the soluble forms of Aβ peptides and not due to aggregated forms of Aβ peptides (proto-fibrils and fibrils), we investigated the extent of Aβ peptide fibrillization under assay conditions by Thioflavin-T (ThT) aggregation assay (Kumar et al., 2016b). Even at the highest concentration points used in our experiments for Aβ40 (800 ng/mL) and Aβ42 (100 ng/mL), no significant differences in the ThT fluorescence intensity were observed between wells containing Aβ peptides and control wells with no Aβ (**Figure 5D**).

Thus, we may conclude that mainly the soluble forms of Aβ peptides were responsible for elevating the catalytic efficiency of ChAT under our assay conditions.

Overall, our detailed *in vitro* enzyme kinetic analyses indicate a possible direct physical interaction between Aβ peptides and ChAT protein.

## Discussion

To the best of our knowledge, this is the first report providing direct evidence for a molecular interaction between Aβ peptides and the core-cholinergic enzyme, ChAT, responsible for the biosynthesis of the neurotransmitter ACh. In this study, we performed detailed *in vitro* biochemical kinetic analyses using a novel real-time fluorometric kinetic assay for ChAT, in combination with ThT fluorescence Aβ fibrillization assay.

Here, we show that soluble forms of Aβ, in particular Aβ42 peptides, are able to enhance at physiological concentrations the catalytic efficiency of human rChAT protein.

**FIGURE 6:**
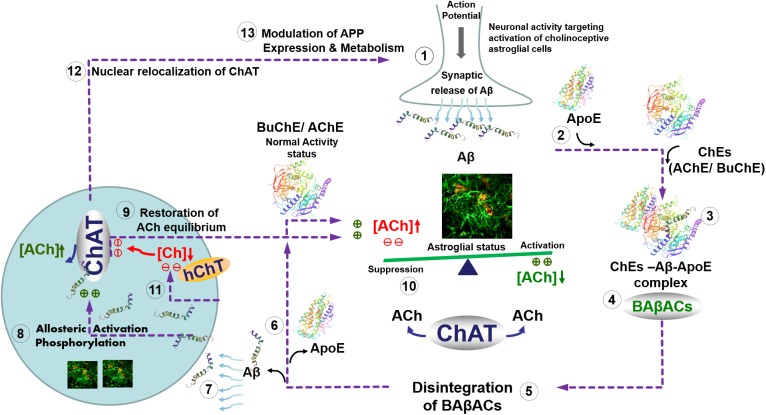
Hypothetical regulatory pathway of the synaptic and extrasynaptic cholinergic signaling by Aβ peptides. (**1**) Neuronal activity demanding activation of cholinoceptive astroglial cells initiate an action potential-synchronized release of the intraneuronal Aβ peptides (Cirrito et al., 2005), e.g., from cholinergic neurons (Baker-Nigh et al., 2015) into the interstitial fluid. (**2**) Simultaneous release of ApoE by astroglia may facilitate the interaction of the released Aβ peptides with the soluble cholinesterases (ChEs) (**3**), leading to the formation of BAβACs. Binding of Aβ peptides to the ChEs leads to an increased intrinsic catalytic rate of these enzymes, plausibly through allosteric modulation of their tertiary structures. (**4**) The hyperfunctional BAβACs will then effectively lower the extrasynaptic acetylcholine (ACh) and thus shifts its equilibrium state, which is maintained by the soluble ACh-synthesizing enzyme choline acetyl-transferase (ChAT) (Vijayaraghavan et al., 2013). The lowered ACh will result in a heightened functional status of astroglial cells, allowing them to perform the intended task. (**5**) Cellular re-uptake of Aβ peptides will then reduce the levels of Aβ peptides, leading to the disintegration of BAβACs. (**6**) The enzymatic activity of ChEs will then return to a relatively dormant state (Darreh-Shori et al., 2011a). (**7**) Cellular reuptake of Aβ by astrocytes and cholinergic neurons will simultaneously augment intracellular ACh production, (**8**) either through direct allosteric activation of ChAT by Aβ peptides (as shown in the current study) and/or by inducing a hierarchical phosphorylation of ChAT (Dobransky et al., 2003). (**9**) This will lead to the additional release of ACh by astrocytes and cholinergic neurons. (**10**) The additional release of ACh together with *in situ* resynthesis of ACh by soluble ChAT, effectively normalize the initial steady-state balance of ACh levels, and thereby allow the astroglial cells to regain their initial activity status (Malmsten et al., 2014). (**11**) The increasing intracellular Aβ will then inhibit reuptake of choline into the cells by inhibiting the high-affinity choline transporter (Cuddy et al., 2017) restricting the amount of substrate (choline) for production of intracellular ACh by the hyperactivated ChAT. (**12**) Increased levels of intracellular Aβ will also induce translocation of a membrane-bound 82kDa variant of ChAT to the nucleus (Winick-Ng et al., 2016) where (**13**) it will modulate gene expression and metabolism of the amyloid precursor protein (APP) (Albers et al., 2014). This will restore the overall balance of ACh homeostasis in the brain (**1**). In Alzheimer’s disease, this ACh homeostasis is disturbed via reduced cellular Aβ reuptake by abnormal levels of ApoE protein (Darreh-Shori et al., 2011b), which forms strong complexes with Aβ peptides (Kumar et al., 2016a). This not only reduces the clearance of Aβ but also forces abnormal accumulation of BAβACs leading to reduced extracellular ACh and hyperactivation of astroglial function that will gradually give rise to a detrimental microenvironment in the brain, leading to the development of Alzheimer’s disease.

Intriguingly, we have previously reported that one of the physiological functions of Aβ peptides could be allosteric modulation of ACh level through the formation of a hyperactive ACh-degrading complex with cholinesterases and ApoE protein, termed BAβACs (Kumar et al., 2016b). The current findings indicate that the role of Aβ peptides in the modulation of the cholinergic signaling is more complex and bidirectional. A plausible hypothetical mechanistic pathway for this complex bidirectional interaction between Aβ and cholinergic machinery is summarized schematically in the **Figure 6**. In other words, we hypothesize that Aβ peptides are on demand able to enhance or reduce the synaptic and/or extrasynaptic cholinergic signaling at several points of interactions, e.g., by allosteric activation of ChAT or the cholinesterases through BAβACs. In addition, the same pathway may allow the cholinergic system to regulate the gene expression and processing of APP. In addition, this hypothetical pathway explains another reported direct interaction of Aβ peptides with the high-affinity choline transporter, that seemingly reduces cellular choline reuptake (Cuddy et al., 2017). This picture fits also with the reported preferential intracellular accumulation of Aβ peptides in the basal forebrain cholinergic neurons, which seems to occur regardless of age or disease status (Baker-Nigh et al., 2015). The same pathway also accounts for reports suggesting that a nuclear form of ChAT decreases the processing of APP and release of Aβ by altering APP gene expression and reducing BACE1 protein levels (Resendes et al., 1999; Albers et al., 2014; Winick-Ng et al., 2016). Thus, the current findings advance our previous hypothesis (Vijayaraghavan et al., 2013; Kumar et al., 2016b).

Surprisingly, Aβ40 inhibited ChAT in some experiments, while in others it enhanced its activity. Given that the concentration of Aβ40 is about 10 folds higher than Aβ42 (at least in CSF), it is possible that Aβ40 is regulating the Aβ42-enhancement of ChAT activity by competing for the binding site. Complementary *in silico* docking and molecular dynamic simulation analyses should be able to provide some insights in this regard. Indeed, our preliminary *in silico* data supports this notion (unpublished data), which will be upon completion published elsewhere.

Altogether, these observations indicate the presence of a mutual physiological regulatory loop between production, intracellular storage, and release of Aβ and the function of the cholinergic system. The understanding about the existence of this loop will provide guiding clues for the link between the early and selective degeneration of the central cholinergic system and the other pathological hallmarks of AD, i.e., the intracellular and extracellular deposition of tau and Aβ. This in turn emphasizes the need for further mechanistic studies aimed at deciphering and understating the key pathological events altering the proper functioning of this regulatory Aβ-cholinergic loop in AD-type dementias. These observations may also explain why studies on Aβ pathology outside the context of this functional loop have up-to-date resulted in unsuccessful therapeutic outcomes, as is evident by the failure of all clinical trials on drugs targeting Aβ metabolism, clearance and/or deposition. This may also be one of the reasons for the lack of correlation between the *in vivo* Aβ-PET imaging assessments and the clinical feature and status of the patients.

One of the limitations in the current study was the use of recombinant protein and peptides rather than tissue extracts. Nonetheless, this was essential since the reported ChAT assay is currently not compatible with tissue homogenates or other biological samples containing large amount of thiol-compounds as they are interacting with the assay’s detecting agent CPM. However, to somewhat compensate for this limitation and to mimic the physiological system, we used relatively a high concentration of gelatin. It should be noted here that the bovine serum albumin also interfered with the detection reagent CPM.

## Conclusion

In conclusion, we present here for the first time a direct effect of Aβ peptides on the catalytic efficiency of the key cholinergic enzyme ChAT, using detailed *in vitro* enzyme kinetics. Together with previous findings concerning Aβ interaction with ACh-degrading enzymes and formation of BAβACs (Kumar et al., 2016b), and with HACU transporter, our current results suggest a much broader bi-directional role of Aβ peptides in regulating ACh homeostasis, and thereby cholinergic signaling. Overall, the findings warrant further investigation toward a deeper understanding of the pathophysiological interrelationship between Aβ peptides and cholinergic function in neurodegenerative disorders, in particular Alzheimer’s disease.

## Author Contributions

The concept and design of the study were done by TD-S. The enzyme kinetics was done and the first draft of the manuscript was written by EL and AK. RK and CL provided critical input and suggestions in writing the manuscript. All the authors read and approved the final draft of the manuscript.

## Conflict of Interest Statement

The authors declare that the research was conducted in the absence of any commercial or financial relationships that could be construed as a potential conflict of interest.
